# Inverse Resolution Limit of Partition Density and Detecting Overlapping Communities by Link-Surprise

**DOI:** 10.1038/s41598-017-12432-1

**Published:** 2017-09-29

**Authors:** Juyong Lee, Zhong-Yuan Zhang, Jooyoung Lee, Bernard R. Brooks, Yong-Yeol Ahn

**Affiliations:** 1 Laboratory of Computational Biology, National Heart, Lung, and Blood Institute (NHLBI), National Institutes of Health (NIH), Bethesda, MD 20852 USA; 2 Department of Chemistry, Kangwon National University, 1 Kangwondaehak-gil, Chuncheon, 24341 Republic of Korea; 3 School of Statistics and Mathematics, Central University of Finance and Economics, Beijing, China; 4 Center for Complex Networks and Systems Research, School of Informatics, Computing, and Engineering, Indiana University Bloomington, Bloomington, IN 47408 USA; 5 Center for In Silico Protein Science, School of Computational Science, Korea Institute for Advanced Study, Seoul, 02455 Korea; 6 School of Computational Sciences, Korea Institute for Advanced Study, Seoul, 02455 Republic of Korea

## Abstract

Finding overlapping communities of complex networks remains a challenge in network science. To address this challenge, one of the widely used approaches is finding the communities of links by optimizing the objective function, partition density. In this study, we show that partition density suffers from inverse resolution limit; it has a strong preference to triangles. This resolution limit makes partition density an improper objective function for global optimization. The conditions where partition density prefers triangles to larger link community structures are analytically derived and confirmed with global optimization calculations using synthetic and real-world networks. To overcome this limitation of partition density, we suggest an alternative measure, Link Surprise, to find link communities, which is suitable for global optimization. Benchmark studies demonstrate that global optimization of Link Surprise yields meaningful and more accurate link community structures than partition density optimization.

## Introduction

Finding community structure is essential in understanding the mesoscale organizations of complex networks. Conventional paradigms assign nodes into groups that optimize an objective function, which measures how meaningful the grouping is^1^. Community detection methods are classified into two broad categories based on whether they allow a node to be included in multiple communities (overlapping communities) or not (disjoint communities). For the latter, one of the most widely used objective functions is modularity^2^. It measures the difference between the number of links between the nodes in the same community and the expected number of links when the network is randomly re-wired. Various optimization methods have been suggested to find the global maximum of modularity^1,3–5^. Although modularity has been widely used to analyze various social and biological networks^6,7^, several drawbacks have been found^1,8^. One of the most significant problems is so-called “resolution limit”^9–13^. As a network becomes larger, the expected number of links within a group decreases, eventually leading to the situation where even merging two distinct complete cliques is better than keeping them separated. Thus, small but meaningful communities in a large network may not be detectable with modularity.

Meanwhile, it has been argued that communities overlap pervasively in many real-world networks^14,15^. For example, in social networks, each person participates in multiple social groups. In biological networks, a protein may play diverse roles in multiple biological processes^6,7,16,17^. Among many overlapping community detection methods that have been suggested^14,15,18–26^, here we focus on the “link community” paradigm, where the communities are redefined as sets of links (edges) rather than nodes^15,19^. This framework provides a clean way to handle pervasive overlaps between communities because a node can be associated with multiple links included in different communities. Identifying communities of *links* in a graph is equivalent to identifying disjoint communities of *nodes* in the “line graph” of the original graph^15,19,27,28^.

**Figure 1:**
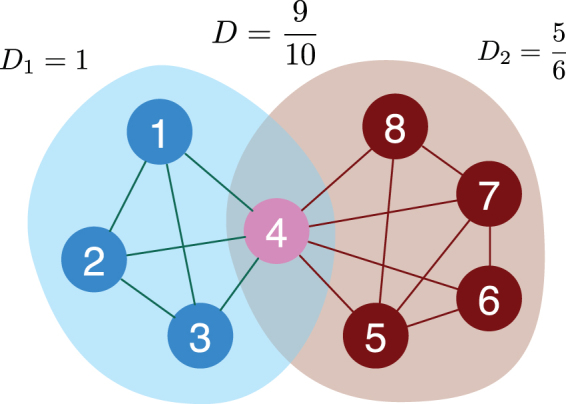
Definition of partition density. A toy example shows how partition density is calculated. The local partition density of the blue nodes *D*_1_ is one because it is a clique, while that of the red nodes *D*_2_ is less than one. The total partition density *D* of the community structure is the weighted sum of two local partition densities, 0.9.

To assess the quality of link communities of a network, “partition density” was proposed as an objective function for link communities^15^. For an undirected and unweighted network, we assume a disjoint partition of links $C=\{{C}_{1},\ldots ,{C}_{{n}_{c}}\}$ where *n*_*c*_ is the number of link communities. The local partition density of a link community *C*_*α*_ is:

1 $${D}_{\alpha }=\frac{{m}_{\alpha }-{\underline{m}}_{\alpha }}{{\overline{m}}_{\alpha }-{\underline{m}}_{\alpha }},$$

where *m*_*α*_ is the number of links in the community *C*_*α*_, ${\underline{m}}_{\alpha }=({n}_{\alpha }-\mathrm{1)}$ and ${\overline{m}}_{\alpha }=\frac{{n}_{\alpha }({n}_{\alpha }-\mathrm{1)}}{2}$ are the minimum and maximum possible numbers of links between the induced nodes that the links in *C*_*α*_ touch, assuming that the nodes in *C*_*α*_ are connected, and *n*_*α*_ is the number of the induced nodes. If the induced nodes are not connected, *D*_*α*_ is set to 0. The partition density of the entire network is:

2 $$D=\sum _{\alpha \mathrm{=1}}^{{n}_{c}}\frac{{m}_{\alpha }}{M}{D}_{\alpha },$$

where *M* is the number of links in the network^15^. Fig. 1 shows a toy example that illustrates how partition density is calculated. By employing hierarchical clustering and Jaccard index-based link similarity measure, a previous study argued that partition density could identify meaningful communities evaluated by the similarity of the metadata of the nodes^15^. Additionally, it was suggested that partition density is free from the problem of resolution limit observed in modularity because partition density only uses local information^9,15^.

Because partition density was effective in previouse studies, it is natural to ask whether it can be used as an objective function for direct global optimization, as in the case of modularity^3,6,7^. However, as we will show below, partition density heavily suffers from its preference towards triangles since it measures pure local density without incorporating any statistical null model. We call this limitation an inverse resolution limit. Here it is clearly demonstrated that a strong preference towards small communities is too critical to use partition density as an objective function for direct global optimization. Global optimization of partition density simply identifies many 3-cliques (triangles) in a network. We show when exactly triangles are favored or not by using toy models and a systematic classification of triangles based on their connectivity. Our analysis demonstrates that larger link communities are favored only in highly limited conditions. To address this limitation, we suggest an alternative approach that formulates link community detection as a global optimization problem.

## Results

**Figure 2:**
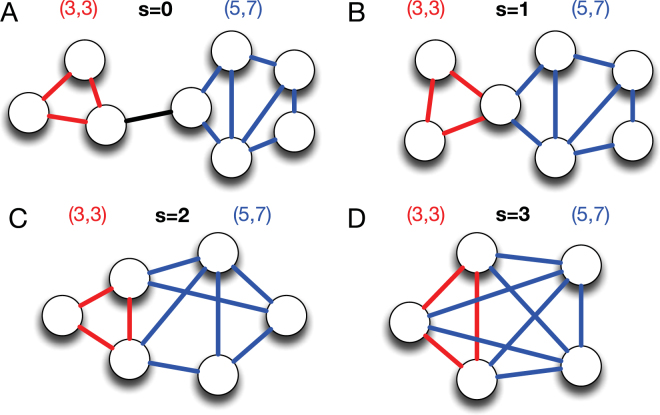
Four possible cases where a triangle is connected with a larger link community. Schematic representations of a triangle (red) and another link community (blue) with *n* nodes and *m* edges sharing *s* nodes, (**A**) *s* = 0, (**B**) *s* = 1, (**C**) *s* = 2, and (**D**) *s* = 3. Here, the number of nodes and edges of the other link community is set to 5 and 7, (*n*, *m*) = (5, 7).

In this section, we examine partition density’s strong preference towards triangles in detail. Without loss of generality, we can assume that there is one triangle *T* in a local link community *C*. Let us assume that *T* shares *s* nodes with the rest of the link community *R* containing *n* nodes and *m* edges. There are four possible choices for the value of *s*, which is shown in Fig. 2.

By definition, a partition density *D* of the community *C* is:

3 $$D=\frac{2}{M}\frac{(m+\mathrm{3)}(m-n+s+1)}{(n-s+\mathrm{1)(}n-s+\mathrm{2)}}$$

where *M* is the total number of links in the whole network, *n* + 3 − *s* and *m* + 3 are the numbers of nodes and links included in the community *C*, respectively.

The partition density *D*_*T*_ and *D*_*R*_ of the triangle *T* and the subnetwork *R* are

4 $${D}_{T}=\frac{3}{M},$$

and

5 $${D}_{R}=\frac{2}{M}\frac{m(m-n+\mathrm{1)}}{(n-\mathrm{1)(}n-\mathrm{2)}},$$

respectively.

The condition where the separation of triangle *T* is preferred can be determined by solving the following inequality:

6 $${\rm{\Delta }}D={D}_{1}+{D}_{2}-D$$

7 $$=\frac{1}{M}\{3+\frac{2m(m-n+\mathrm{1)}}{(n-\mathrm{1)(}n-\mathrm{2)}}-\frac{\mathrm{2(}m+\mathrm{3)(}m+s-n+\mathrm{1)}}{(n-s+\mathrm{2)(}n-s+\mathrm{1)}}\} > \mathrm{0,}$$

If Δ*D* is negative, the triangle *T* and its neighboring link community *R* will merge into one community. Otherwise, they prefer to be separated.

When *s* = 0,

8 $${\rm{\Delta }}D=\frac{1}{M}[3+(m-n+\mathrm{1)}\{\frac{2m}{(n-\mathrm{1)(}n-\mathrm{2)}}-\frac{\mathrm{2(}m+\mathrm{3)}}{(n+\mathrm{1)(}n+\mathrm{2)}}\}]$$

If *m* is replaced with the minimum number of links between *n* nodes, *n* − 1, Δ*D* = 3/*M*, which is positive. Because Δ*D* is an increasing function of *m*, Δ*D* is always positive. Therefore, the separation of a triangle is *always preferred* when there is no shared node.

Similarly, if *s* = 1,

9 $${\rm{\Delta }}D=\frac{1}{M}\frac{\mathrm{(4}n-\mathrm{2)}{m}^{2}-\mathrm{(8}{n}^{2}-18n+\mathrm{10)}m+3{n}^{3}-15{n}^{2}+24n-12}{(n-\mathrm{2)(}n-\mathrm{1)}n(n+\mathrm{1)}},$$

which is a quadratic function of *m* whose minimum is located at (4*n* ^2^ − 9*n* + 5)/(4*n* − 2). Because *n* > 2, the denominator (*n* − 2)(*n* − 1)*n*(*n* + 1) is positive, and the coefficient of *m* ^2^, 4*n* − 2, is also positive. Thus, if *m* is larger than (4*n* ^2^ − 9*n* + 5)/(4*n* − 2), Δ*D* is a monotonically increasing function of *m* with a fixed *n* value. By definition, the minimum of *m* is *n* − 1, which is larger than (4*n* ^2^ − 9*n* + 5)/(4*n* − 2). If *m* is replaced by *n* − 1, Δ*D* is positive. Therefore, the separation of a triangle is *always preferred*.

If *s* = 2,

10 $${\rm{\Delta }}D=\frac{1}{M}\frac{4{m}^{2}+\mathrm{(24}-14n)m+3{n}^{3}-3{n}^{2}-24n+36}{(n-\mathrm{2)(}n-\mathrm{1)}n}\mathrm{.}$$

Similar analysis shows that, with a fixed *n* value, Δ*D* is a monotonically increasing function of *m* if *m* is larger than (7*n* − 12)/4. If *m* is replaced by (7*n* − 12)/4, Δ*D* is positive except for the case of *n* = 3. If *n* = 3, Δ*D* is negative when *m* has its minimum value *n* − 1 = 2. Δ*D* keeps decreasing as *m* increases until *m* = (7*n* − 12)/4 = 9/4. After *m* = 9/4, Δ*D* increases and becomes positive again when *m* = 3. Hence Δ*D* is always positive except the case of *n* = 3 and *m* = 2.

**Figure 3:**
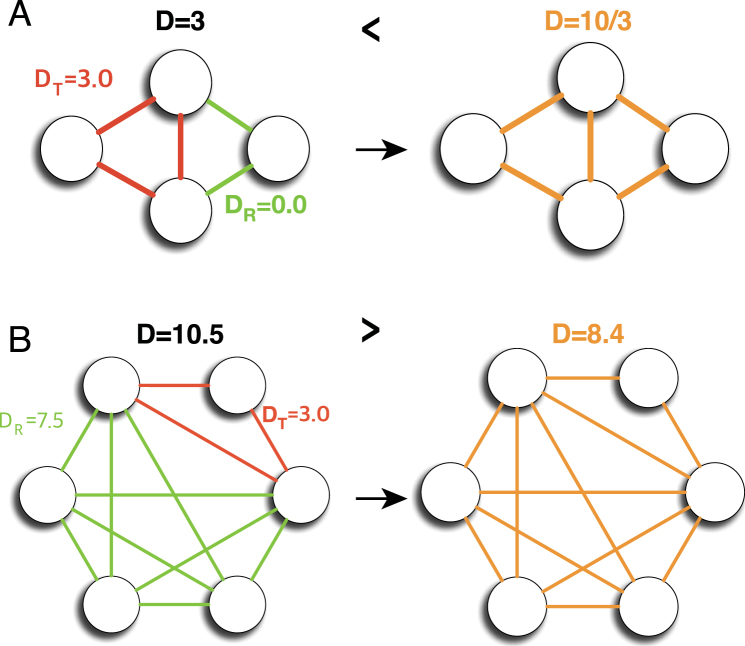
Link communities with two shared nodes. Examples of link communities that are (**A**) not separable and (**B**) separable when two nodes are shared between a triangle and the rest of link community, *s* = 2.

This result clearly shows why triangles are preferred by the current definition of partition density. It indicates that, for a given link community consisting of [four of more nodes] if there exists *an independent triangle* that contains a node that is not connected with the rest of nodes in the same community, separating the triangle is *always preferred*. Figure 3 shows the examples of *s* = 2 cases. In Fig. 3A, the partition density of the green triangle is 3, and the rest of links form a linear community with a partition density of 0, which results in the total partition density of 3. Here, the denominator *M* in equation 2 is omitted since it is a constant. However, when the two link communities are merged, the partition density becomes 10/3, which makes the separation of the triangle unfavorable. On the contrary, on the right side of Fig. 3B, the entire link community consists of 6 nodes and 12 edges and contains an independent triangle. The partition density of the community is 8.4. However, if the independent triangle (colored in red in Fig. 3) is separated; the sum of partition densities becomes 10.5, which makes the separation of the triangle favorable.

If *s* = 3, there is no independent triangle in a link community, i.e., all nodes share at least three links with others in a community. In this case, Δ*D* can be written as below:

11 $${\rm{\Delta }}D=\frac{1}{M}\frac{\mathrm{3(}{n}^{2}-n-4m-\mathrm{6)}}{(n-\mathrm{1)(}n-\mathrm{2)}},$$

which is a linear function of *m*. Δ*D* is negative if the following condition is satisfied:

12 $$m > \frac{1}{4}({n}^{2}-n-\mathrm{6).}$$

Thus, a link community with *n* nodes and *m* links is non-separable if the following condition is satisfied:

13 $$m > \frac{1}{4}({n}^{2}-n-\mathrm{6)}+3.$$

**Figure 4:**
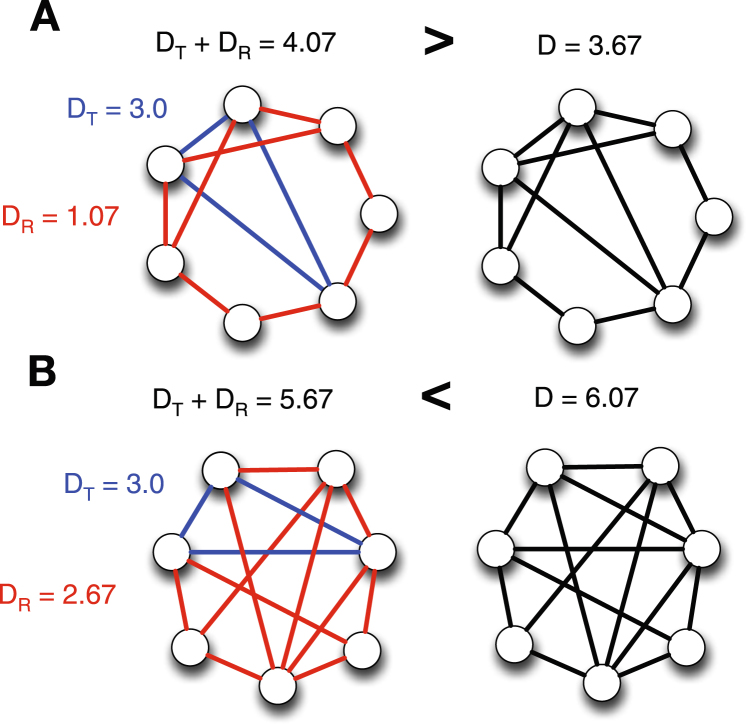
Link communities with three shared nodes. Examples of link communities that have no independent triangle (*s* = 3). In example (**A**), the separation of a triangle is preferred, while example (**B**) is not preferred.

In other words, if equation 13 is not satisfied, a link community is separable although there is no independent triangle in it. Two examples with three shared nodes, *s* = 3, are shown in Fig. 4. The first example does not satisfy equation 13 (Fig. 4A). Thus it prefers to be separated although there is no independent triangle. The partition density of the merged link community is 3.67, while the sum of partition densities of two separated link communities is 4.07. On the other hand, the second example satisfies equation 13 (Fig. 4B). The sum of partition densities of separated link communities, 5.67, is smaller than that of the merged link community, 6.07. Thus the separation of a triangle is not preferred.

**Figure 5:**
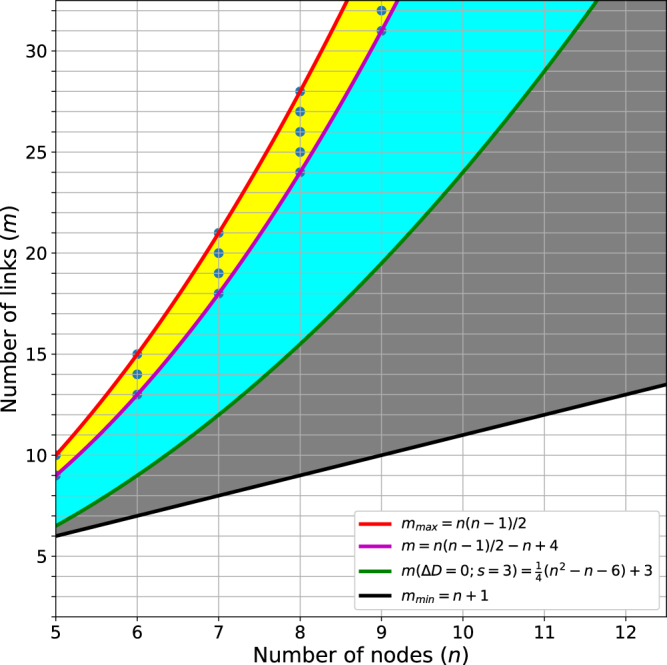
A condition for separation of a triangle. The region plot shows the condition where the separation of a triangle is not preferred (yellow), conditionally (cyan), and always preferred (gray). The red line represents the maximum number of links that can be formed with *n* nodes. The magenta line corresponds to the minimum number of links that the separation of a triangle is impossible. The green line represents the solution Δ*D* = 0 when *s* = 3. The black line represents the minimum number of links to form a link community including a triangle. The blue dots correspond to the conditions that a link community is non-separable.

Based on these results, we can define the condition that a link community is non-separable: no independent triangle exists and equation 13 is satisfied. When doesn’t a link community have an independent triangle? The maximum number of links that has an independent triangle can be found when a link community that has only one node that is connected with two direct neighbors while the rest of nodes are fully connected to each other. If one additional link is added in this link community, all nodes must have at least three links, excluding the existence of an independent triangle. This condition is equivalent to removing *n* − 3 links from *n*-clique,

14 $$m=\frac{n(n-\mathrm{1)}}{2}-(n-\mathrm{3),}$$

which is always larger than equation 13 (Fig. 5). Therefore, if a link community with *n* nodes has more than *n*(*n* − 1)/2 − (*n* − 3) links, the community is not separated.

In summary, based on partition density, a link community including five or more nodes is favored only when it satisfies equation 13 and does not have an independent triangle. If there is an independent triangle in a link community, the triangle prefers to be separated from the community. It is guaranteed that highly cliquish link communities satisfying equation 14 remain intact. In other words, the condition where a link community remains intact under partition density optimization is extremely limited. This indicates that the direct global optimization of partition density yields mostly triangles with few larger link communities, failing to identify “meaningful communities” that are commonly conceptualized.

## Numerical Simulations

To identify how this triangle preference of partition density affects community detection in actual networks, we performed global optimizations of partition density using the conformational space annealing algorithm (CSA). The CSA algorithm has been successfully applied to global optimization of modularity^3^ as well as various global optimization problems^29–36^. The CSA global optimization of modularity^3^ is modified to optimize partition density. Two classes of synthetic networks are used to evaluate the triangle preference of partition density: the Girvan-Newman (GN)^37^ and the Lancichinetti-Fortunato-Radicchi (LFR)^38^ networks.

**Figure 6:**
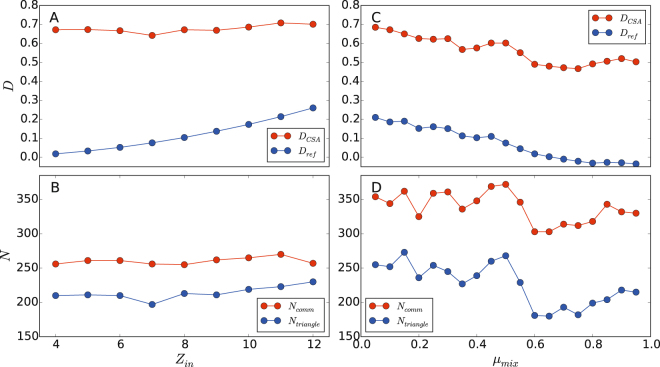
Global optimization of partition density. Optimized partition density and the estimated number of communities by optimization of partition density on the GN and LFR benchmark networks. Subplot **A** and **C** plot the optimized (*D*_*CSA*_) and reference (*D*_ref_) partition densities versus *Z*_in_ and *μ*_mix_ values. Subplot **B** and **D** plot the numbers of all identified link communities (*N*_*comm*_) and triangles (*N*_*triangle*_) versus *Z*_in_ and *μ*_mix_ values.

For the GN networks, we compare optimized *D* (*D*_opt_) values using CSA with the reference *D* (*D*_ref_) value, which is calculated from the pre-defined node-community structure. To calculate the *D*_ref_, all intra-node-community edges of a node-community are considered as the same link community, and inter-node-community edges are ignored. For all GN networks, the *D*_opt_ values are much higher than the *D*_ref_ values (Fig. 6A). The *D*_opt_ values are almost identical for all GN networks, around 0.7, while the *D*_ref_ value monotonically decreases from 0.23 to 0.03 as the community structure of GN network becomes weaker. We also count the numbers of triangles and all link communities from the CSA results (Fig. 6B). For all the GN networks, around 260 link communities are detected via *D*-optimization and, among them; around 220 link communities are triangles on average. In addition, the number of triangles increases as *Z*_in_ increases, suggesting that highly modular networks may suffer more from the inverse resolution limit of *D*. These results show that the global optimization of *D* leads to a significantly different community structure from the reference community due to the triangle preference of *D*.

The benchmark results of the LFR networks show similar trends with those of the GN networks. A comparison of *D*_opt_ and *D*_ref_ values demonstrates that there is a large gap between two values regardless of *μ*_mix_, and both *D* values decrease as networks become less modular, a larger *μ*_mix_ (Fig. 6C). The inverse correlation between *D* and *μ*_mix_ shows that *D* is correlated with the degree of modularity. However, as shown in the GN networks, community structures with high *D* values do not correspond to the reference community structure. From Fig. 6D, it is identified that about 2/3 of detected link communities via *D*-optimization are triangles, and more triangles are detected in the networks with a strong sense of community, *μ*_mix_ < 0.5, than the networks without community, *μ*_mix_ > 0.5.

We also perform *D*-optimization of several popular real-world benchmark networks and compare the numbers of all communities and triangles (Table 1). For all real-world benchmark networks, more than half of detected link communities by *D*-optimization are triangles. This indicates that the inverse resolution limit of partition density is universal regardless of networks.

**Table 1:** The number of triangles and the total number of link communities of real world networks obtained with the global optimization of partition density.

Dataset	# Triangle communities	# Communities
Karate	18	25
Dolphin	30	51
Lesmis	26	44
Political books	83	120
Football	109	168
Netscience_main	98	200
*C. elegans*	297	512
Jazz	562	772
*E. coli*	1466	2184

## Alternative objective function for link communities: Link-Surprise

In previous sections, the limitation of partition density as an objective function for community detection is clearly demonstrated. To address this limitation, we suggest a new objective function to find meaningful link communities by using a random graph as a null model where all pairs of nodes have the equal probability to be connected. For a random network with *n* nodes and *m* edges, the probability to find a link community consisting of *k* nodes and at least *l* edges by chance is given by a cumulative hypergeometric distribution^39,40^:

15 $$P(k,l;n,m)=\sum _{j=l}^{min(m,K)}\frac{(\begin{array}{c}K\\ j\end{array})(\begin{array}{c}N-K\\ m-j\end{array})}{(\begin{array}{c}N\\ m\end{array})},$$

where *K* and *N* are the maximum numbers of links between *k* and *n* nodes, which are *k*(*k* − 1)/2 and *n*(*n* − 1)/2, respectively. On a similar note, the Surprise measure was suggested to find node communities^39,40^. The difference between our approach and the original Surpise measure is that our approach measures the probability of formation of a *local* community defined by a group of links, but Surprise calculates the probability of formation of a *whole* node community structure of a network. Thus, we call our measure Link-Surprise *S*. Since the absolute scale of *P*-value depends on the density of a network, it should be normalized to be a general objective function. To address the normalization issue, the original *P*-value is divided by the *P*-value of a given link community with that of a reference link community corresponding to the smallest meaningful link community. In this work, we used a linear chain of two connected links as the reference community. The Link-Surprise of a single link community is defined as:

16 $$r(k,l;n,m)=P(k,l;n,m)/P({k}_{{\rm{ref}}},{l}_{{\rm{ref}}};n,m\mathrm{).}$$

This ratio estimates the likelihood ratio of forming a given link community compared to a reference community. By using this ratio, the total Link-Surprise of link communities of a network is defined as the logarithm of the product of the Link-Surprise of all link communities:

17 $$S=-\mathrm{log}\,\prod _{i\mathrm{=1}}^{{N}_{{\rm{c}}}}{r}_{i}({k}_{i},{l}_{i};n,m)$$

18 $$\quad \quad =-\sum _{i\mathrm{=1}}^{{N}_{{\rm{c}}}}\,\mathrm{log}\,P({k}_{i},{l}_{i};n,m)-{N}_{{\rm{c}}}\,\mathrm{log}\,P({k}_{{\rm{ref}}},{l}_{{\rm{ref}}};n,m),$$

where *i* is a link community index, *N*_c_ is the number of link communities, and *r*_*i*_ is the ratio of *P*-value of a link community *i*. The last term in equation 18 shows that the normalization using the reference link community reduces the number of link communities. Note that the definition of a reference community may vary and plays the role of a resolution parameter for link community detection.

## Global optimization of Link-Surprise

**Figure 7:**
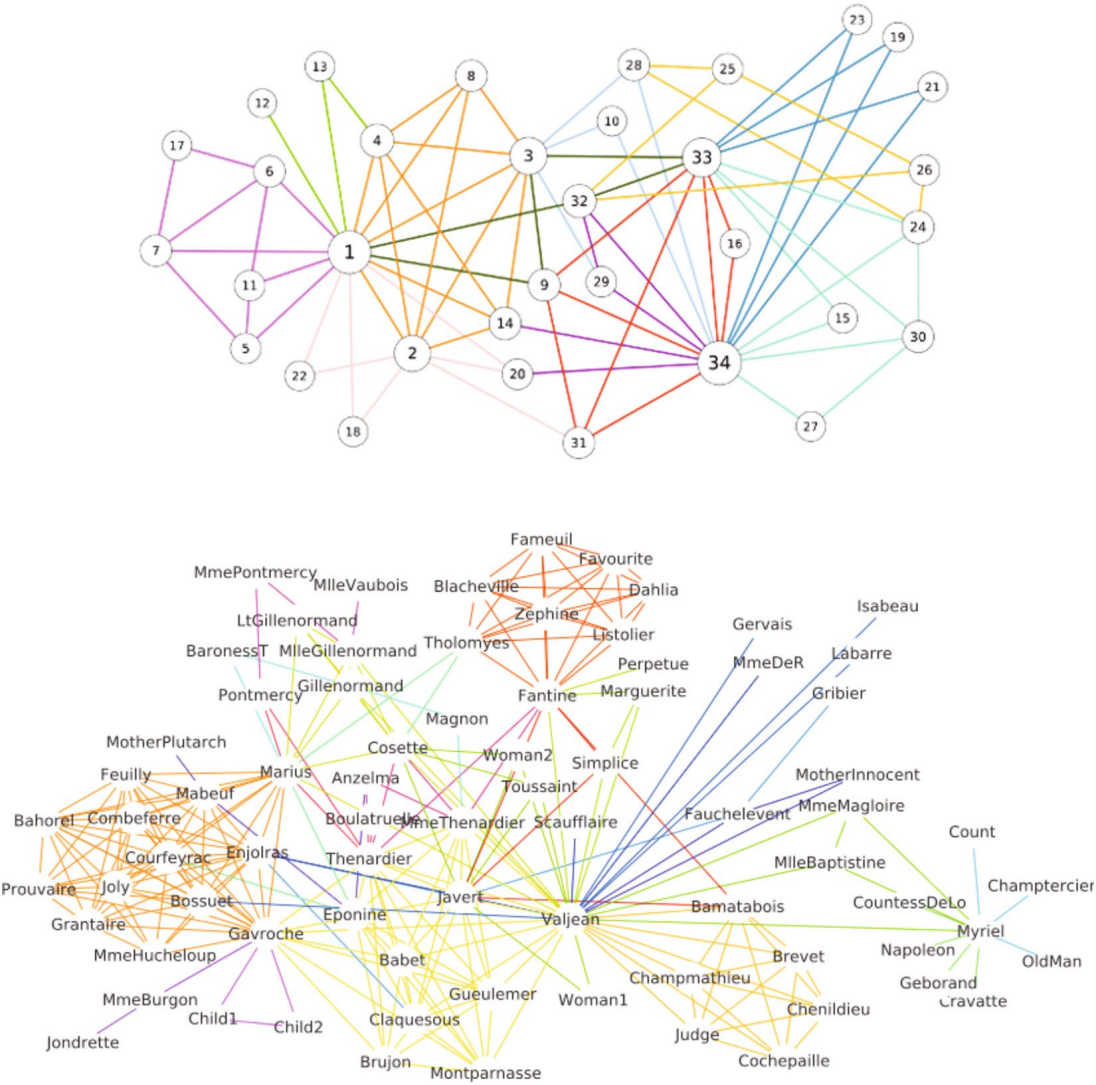
The link communities of the karate and les miserable networks. The link community structures of the (upper) karate and (lower) les miserable networks are determined by the global optimization of the Link-Surprise. The links are colored by their link community membership.

To assess the performance of Link-Surprise, the link communities of the karate and les miserable networks were detected via the global optimization of the Link-Surprise (Fig. 7). The results show that the direct global optimization of the Link-Surprise leads to meaningful link communities, which are larger than a triangle and densely interconnected.

The qualities of community detection results were evaluated by calculating the normalized mutual information (NMI) values for overlapping communities^18^ between the obtained and the reference communities [using the LFR networks]. The obtained NMI values are adjusted by subtracting the average NMI values of randomly shuffled communities while preserving the number of communities to remove the artifact caused by the number of communities^41^. For comparison, community detection of line graphs^19,28^ and clique percolation (CFinder)^14^ approaches were also performed. A triangle was used as a reference clique for the CFinder calculations. For the community detection of line graphs, modularity optimization^3^ and Infomap^42^, approaches were employed.

**Figure 8:**
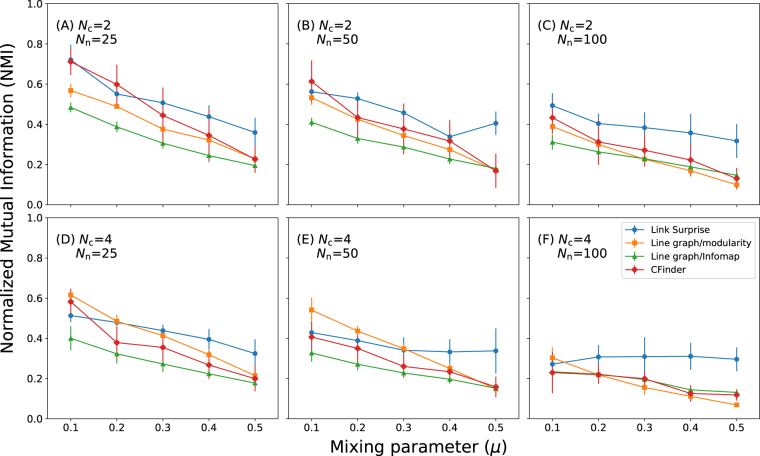
Benchmark results of the Link-Surprise using the LFR networks. The LFR graphs were generated with 200 nodes, an average degree of 10, and the maximum degree of 30. The average NMI values were obtained with 10 iterations by varying mixing parameter *μ*, the number of overlapping nodes *N*_n_, and the number of overlapping communities *N*_c_.

The benchmark results demonstrate that the global optimization of Link-Surprise leads to higher NMI values than the existing methods in most cases (Fig. 8). When the number of overlapping nodes is large, (*N*_n_ = 100), Link-Surprise optimization apparently yields more accurate results than the other methods. Also, as community structures become weaker, (larger *μ* values), Link-Surprise optimization leads to better results than the other methods regardless of *N*_n_ and *N*_o_ values. These results indicate that Link-Surprise can be a promising objective function to detect overlapping communities of large and highly intertwined networks.

## Resolution limits of Link-Surprise

The resolution limit of Link-Surprise is investigated using the ring of cliques in a similar spirit to previous studies^9,12,13^. We assume a network that consists of *r* cliques containing *n*_c_ nodes and two cliques are connected with only one edge to form a ring. Although the network is one of the most modular structures possible, it was shown that many community structure measures favor merging cliques as the network becomes larger, which prevents the detection of small communities^9,12,13^. Here, we will test whether Link-Surprise suffers from the limitation. The total numbers of nodes and edges of the network are *n*_tot_ = *rn*_c_ and *m*_tot_ = *rn*_c_(*n*_c_ − 1)/2 + *r*. To identify the condition where two cliques start to merge, the difference between the Link-Surprise values of two independent cliques and their merged counterpart is calculated:

19 $$\begin{array}{rcl}{\rm{\Delta }}S & = & -2\,\mathrm{log}\,P({n}_{c},{n}_{c}({n}_{c}-\mathrm{1)/2;}{n}_{{\rm{tot}}},{m}_{{\rm{tot}}})+\,\mathrm{log}\,P\mathrm{(2}{n}_{c},{n}_{c}({n}_{c}-\mathrm{1)}\\  &  & +1;{n}_{{\rm{tot}}},{m}_{{\rm{tot}}})-\,\mathrm{log}\,P({k}_{{\rm{ref}}},{l}_{{\rm{ref}}};{n}_{{\rm{tot}}},{m}_{{\rm{tot}}})\end{array}$$

If Δ*S* < 0, Link-Surprise favors merging two cliques into one, corresponding to the resolution limit.

**Figure 9:**
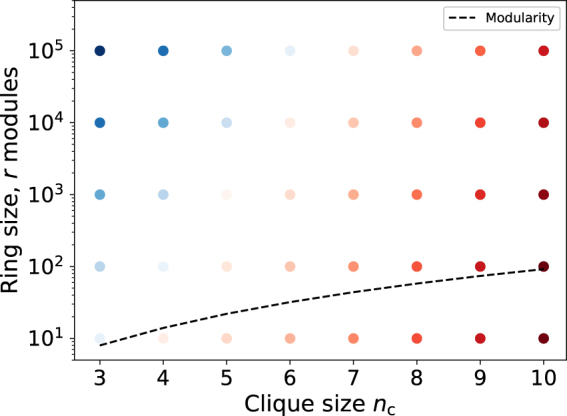
The resolution limit of Link-Surprise. The detectable region of the ring of *m* modules using Link-Surprise and modularity. Each module is a clique with *n*_*c*_ nodes. Red and blue dots represent detectable and undetectable conditions by Link-Surprise, respectively. Darker shade corresponds to a larger absolute difference between the Link-Surprise values of two separate cliques and their merged counterpart. Black dotted line corresponds to the detectable limit of modularity as shown in *r* = *n*_*c*_(*n*_*c*_ − 1) + 2^9^.

The Δ*S* values are calculated with different *n*_*c*_ and *r* values (Fig. 9). It is identified that Link-Surprise also suffers from the resolution limit. For *n*_c_ = 6, two cliques are identified as separate communities when the network has 10^4^ modules, while they become undetectable when the network becomes bigger, 10^5^ modules. Although Link-Surprise is not free from the resolution limit, it is much less severe than modularity whose resolution limit is given by *r* = *n*_*c*_(*n*_*c*_ − 1) + 2^9^. With modularity, two cliques with *n*_c_ = 6 become undetectable when there are only 32 modules. The experiment also shows that Link-Surprise does not suffer from inverse resolution limit. The Δ*S* values become larger as the size of clique increases, which indicates that Link-Surprise favors to form larger cliques than smaller ones.

**Figure 10:**
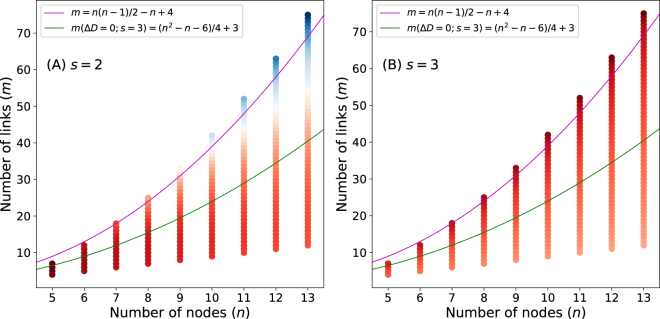
The inverse resolution limit of Link-Surprise. The change of Link-Surprise due to the separation of a triangle is calculated when (**A**) 2 or (**B**) 3 nodes are shared between the triangle and its neighboring link community (see Fig. 2). Blue and red dots correspond to the conditions where the separation of a triangle is favorable and unfavorable, respectively. Magenta and green lines correspond to the conditions where the separation of a triangle is unfavorable and favorable with partition density. The conditions located between the magenta and green lines *conditionally* favors the separation of a triangle and those located below the green line *always* favors the separation of a triangle.

We also investigate whether Link-Surprise suffers from the inverse resolution limit by examining the same examples used for partition density (Fig. 2). The difference between the sum of Link-Surprise values of a triangle *T* and a neighboring link-community *R* and the Link-Surprise of the merged link-community is calculated (Fig. 10). The results show that Link-Surprise is free from the inverse resolution limit. When there is an independent triangle (*s* = 2), the triangle favors to be separated only when the neighboring community *R* is highly cliquish (Fig. 10A). When there is no independent triangle (*s* = 3), a link community is always non-separable with Link-Surprise (Fig. 10B). When a triangle is separated from a link-community with Link-Surprise, the separated link-community *R* becomes more cliquish. Thus, *R* is not divided further. However, with partition density, a sparse link community keeps separated until only highly cliquish link communities remain, which leads to many triangles. In conclusion, the conventional resolution limit of Link-Surprise is much less severe than that of modularity and Link-Surprise is free from the inverse resolution limit.

## Discussion

In this study, we showed that partition density suffers from the strong preference towards a triangle; identifying triangles as separate link communities is preferred in most possible scenarios. Direct global optimization of partition density of the synthetic and the real-world networks resulted in a huge number of triangles. We showed that a triangle contains a node that is connected only to the other two nodes; it always prefers to be separated. The only exception is when four nodes are connected with five links.

One of the reasons for the preference to a triangle is that a difference in local partition density *D*_*α*_ between a triangle and larger cliques or cliquish link communities becomes marginal as a network becomes larger. By definition, a decrease in *D*_*α*_ of a large link community due to a separation of a triangle becomes smaller as the number of induced links increases (equation 1). However, *D*_*α*_ of a separated triangle is always 1.0, which can be large enough to compensate the decreased *D*_*α*_ of the initial link community. Our result raises further questions: how should we handle triangles? Is it more meaningful than a larger cliquish link community? Although a triangle is a clique, it may be too small to extract meaningful information from it and to reduce the complexity of a network efficiently. Thus, a criterion to compare the significance of a triangle and larger cliquish link communities may be necessary.

Considering the strong bias of partition density, how could it work as an objective function for the link clustering method^15^? First, a hierarchical clustering was performed in an agglomerative way to generate the dendrogram of links and detect the community structure of a network based on a threshold that maximizes partition density. With this approach, a formation of triangles is suppressed because clustering is carried out in a way that the size of a cluster only increases by merging the most similar pair of links first, imposing strong constraints on the community structures. Second, the heterogeneity of a network might have play an important role. If the degree distribution of nodes follows a uniform or a Gaussian distribution, many nodes may have similar numbers of links, direct neighbors, which make most pairs of links have similar similarities. If this is the case, many triangles may have been formed due to a high degeneracy of priorities of links for merging. However, many real-world networks are known to be scale-free networks whose degree distributions are highly heterogeneous. The heterogeneity of connectivity leads to a heterogeneous distribution of link similarities, which results in the formation of the hierarchical organization of link communities^15^.

As an alternative objective function to partition density, we introduce Link-Surprise, which measures the probability to form a given link community structure by assuming a random graph-based null model. A higher Link-Surprise indicates that a given community structure is less likely to be formed by chance. The major difference of Link-Surprise from Significance^39,40^, which was suggested for disjoint community detection, is that Link-Surprise is the product of P-values of all local link communities (equation 17), whereas Significance is the single P-value of a given community structure of all nodes. In addition, the concept of the reference link community is introduced in Link-Surprise to facilitate the detection of non-trivial community structures and to enhance the convergence of optimization of Link-Surprise. The Link-Surprise values of link communities whose P-values are larger, i.e., less significant, than the reference are ignored. In this study, a chain of two connected links is used as a reference link community, which is the smallest subgraph of connected links. Practically, this reference community may play a role of a resolution parameter in other community detection methods^1,43–45^. For large networks, using a more complex reference community would enhance efficiency and convergence of global optimization of Link-Surprise.

Unlike partition density, the benchmark simulations demonstrate that the global optimization of Link-Surprise leads to a set of meaningful link communities rather than a set of many triangles (Figs 7 and 8). The benchmarks with the LFR networks show that Link-Surprise optimization yields more accurate overlapping community structures than existing approaches particulary when the number of overlapping nodes *N*_n_ and a mixing parameter *μ* are large (Fig. 8). This indicates that Link-Surprise could be an useful measure to find the community structures of densely connected networks with many overlaps between communities.

## Methods

### Global optimization of partition density

The GN network consists of 128 nodes divided into four node communities of 32 nodes. Each node is connected to the other nodes in the same community with *Z*_in_ links and to nodes in other modules with *Z*_out_ links. Every node has 16 links in total, *Z*_in_ + *Z*_out_ = 16. When *Z*_in_ > 8, each node has more connections within the community than the rest of network and corresponds well to the four pre-defined communities. In the LFR network, the node degrees and community sizes are stochastically assigned to follow a power-law distribution. Links are stochastically connected based on a mixing parameter *μ*_mix_, ranging from 0 to 1. Each node shares a fraction of 1 − *μ*_mix_ of links with the other nodes in the same community, and a fraction of *μ*_mix_ of links with the rest of network. Thus, a community structure becomes weaker as *μ*_mix_ increases, and a community structure in a strong sense exists until *μ*_mix_ < 0.5. In this study, GN networks are generated with *Z*_in_ values ranging from 4 to 12. LFR networks are generated with a degree distribution ranging from 10 to 50 based on a power-law distribution with an exponent of 2. Community sizes are tuned to follow a power-law distribution with an exponent of 1 and range from 10 to 30.

### Global optimization of Link-Surprise

Global optimization calculations of Link-Surprise were performed with the LFR benchmark networks for overlapping communities. The networks were generated with 200 nodes, mixing probabilities *μ*_mix_ of 0.1 and 0.3, the number of overlapping nodes *N*_n_ of 25, 50, and 100, and the numbers of memberships of an overlapping node *N*_o_ of 2 and 4^38^. With each parameter set, ten independent networks were generated, and the link communities were determined by the global optimization of the Link-Surprise using the simulated annealing approach^3,46^.

### Data availability

All relevant data are available from the authors upon request.
